# Assessing the content and quality of GI bleeding information on Bilibili, TikTok, and YouTube: a cross-sectional study

**DOI:** 10.1038/s41598-025-98364-7

**Published:** 2025-04-28

**Authors:** Jingsong Wang, Bingxi Liu, Guang Yang, Yixing Luo, Nonghua Lv, Xu Shu, Zhenhua Zhu, Linlin Liu

**Affiliations:** 1 Department of Gastroenterology, Jiangxi Provincial Key Laboratory of Digestive Diseases, Jiangxi Clinical Research Center for Gastroenterology, Digestive Disease Hospital, The First Affiliated Hospital, Jiangxi Medical College, Nanchang University, Nanchang, Jiangxi China; 2 Department of Gastroenterology, South China Hospital, Medical School, Shenzhen University, Shenzhen, 518116 P. R. China; 3 School of Medicine, Jianghan University, Wuhan, 430056 China

**Keywords:** Gastrointestinal bleeding, Health information, TikTok, Bilibili, YouTube, Online video, Gastrointestinal bleeding, Patient education

## Abstract

Gastrointestinal (GI) bleeding is a significant clinical issue that is commonly discussed on social media platforms. Despite the widespread availability of health information videos, the quality and comprehensiveness of these videos vary greatly, posing potential risks to public health. This study evaluates the content and quality of GI bleeding-related videos on TikTok, Bilibili, and YouTube. A total of 300 GI bleeding-related videos were collected and analyzed from TikTok, Bilibili, and YouTube. The videos were categorized based on the uploader’s background (health professionals vs. non-health professionals). Key metrics such as likes, comments, video duration, and quality scores (GQS, JAMA, and modified DISCERN) were compared. Simultaneously, the content of the videos was analyzed across six aspects: definition, symptoms, risk factors, tests, treatment, and outcomes. A total of 300 GI bleeding-related videos were analyzed, with 196 (65.3%) uploaded by health professionals and 104 (34.7%) by non-health professionals. TikTok videos received significantly more likes and comments but were shorter in duration compared to those on Bilibili and YouTube (*p* < 0.05). The overall quality and reliability of the videos were relatively low, with median DISCERN and GQS scores of 2 (IQR: 2–3) and 3 (IQR: 2–3), respectively. Videos from health professionals exhibited higher reliability and quality, with median DISCERN scores of 3 (IQR: 3–4) and GQS scores of 3 (IQR: 3–4). The overall quality and comprehensiveness of GI bleeding-related videos on social media platforms are unsatisfactory, with many low-quality videos still prevalent. Although videos from health professionals score higher in quality and reliability, their content comprehensiveness is not significantly better than those from non-health professionals. Efforts should be intensified to improve the content and quality of GI bleeding videos, raise public awareness, and promote patient self-management.

**Supplementary Information:**

The online version contains supplementary material available at 10.1038/s41598-025-98364-7.

## Introduction

Gastrointestinal (GI) bleeding is a common medical emergency with a high incidence rate and potential for serious complications^1^. GI bleeding leads to millions of hospitalizations annually, imposing a significant burden on healthcare costs^2–4^. Despite advancements in medical technology and treatment methods in recent years, GI bleeding remains a major challenge in the global health sector^5,6^. Understanding the various aspects of GI bleeding, including its causes, symptoms, and treatments, can aid in better prevention and management of this medical issue, thereby alleviating the strain on patients and healthcare systems^7–9^.

With the rapid development of social media, video content has become a crucial means of information dissemination^10–12^. Many internet users obtain medical information through online video platforms, which not only provide convenient learning channels but also significantly enhance public health knowledge. According to a study by Peng et al. (2014), a substantial portion of health information seekers in China turn to online video platforms for medical advice, with over 60% of respondents indicating that these platforms are their primary source of health information^13–15^. However, as the number of medical videos on these platforms has increased rapidly, the issue of inconsistent quality has become more pronounced. The quality of medical video content on these platforms directly influences viewers’ health decisions and behaviors. High-quality videos can offer accurate and authoritative medical knowledge, helping users better understand complex medical issues and treatment options. In contrast, low-quality videos may spread misinformation, mislead viewers, and potentially have negative impacts on their health^15–17^. Therefore, ensuring the quality of medical videos on online platforms is of utmost importance. For this study, we selected TikTok, Bilibili, and YouTube due to their popularity and widespread use in China and globally. These platforms were chosen to provide a comprehensive overview of GI bleeding-related videos in the Chinese context. Other platforms like Facebook Reels and Instagram Reels were not included due to their limited accessibility in China.

Social media platforms such as Bilibili, TikTok, and YouTube play a significant role in disseminating medical knowledge about gastrointestinal (GI) bleeding^22^. As widely used video-sharing channels, these platforms have become primary sources for the public to obtain health information^14,17,18^. Bilibili is a popular Chinese video-sharing platform that focuses on a wide range of content, including anime, music, dance, technology, and educational videos. It is particularly known for its strong community interaction and user-generated content, making it a significant source of information and entertainment for young audiences in China. YouTube, the world’s largest long-form video sharing platform, boasts over one billion users, although it is inaccessible in China. In contrast, TikTok are among the most popular short video platforms in China, with hundreds of millions of daily active users. These social media platforms, leveraging their vast user bases and rich video content, provide a wealth of information related to GI bleeding^19–21^.However, due to content regulation on these platforms, the searches conducted on Chinese platforms like Bilibili and TikTok could be biased. These regulations may influence the availability and type of content that can be accessed and shared, potentially limiting the comprehensiveness of the information obtained. Despite these limitations, these platforms remain primary sources for the public to obtain health information. Due to differences in public education levels and the limitations of content regulation on these platforms, the dissemination of GI bleeding information varies in quality and can sometimes be misleading. Some videos may contain inaccurate or misleading information, causing confusion and potential risks for patients and the general public^22–24^.

Currently, there is a lack of research specifically analyzing the status of online videos about GI bleeding. Given the growing public interest in GI bleeding, it is crucial to evaluate the effectiveness of these media platforms in conveying information about the condition. Therefore, this study aims to assess the content, reliability, and quality of GI bleeding-related videos available on Bilibili, TikTok, and YouTube, to provide the public with more comprehensive and accurate guidance on obtaining and understanding information about GI bleeding.

## Methods

### Search strategy

**Fig. 1:**
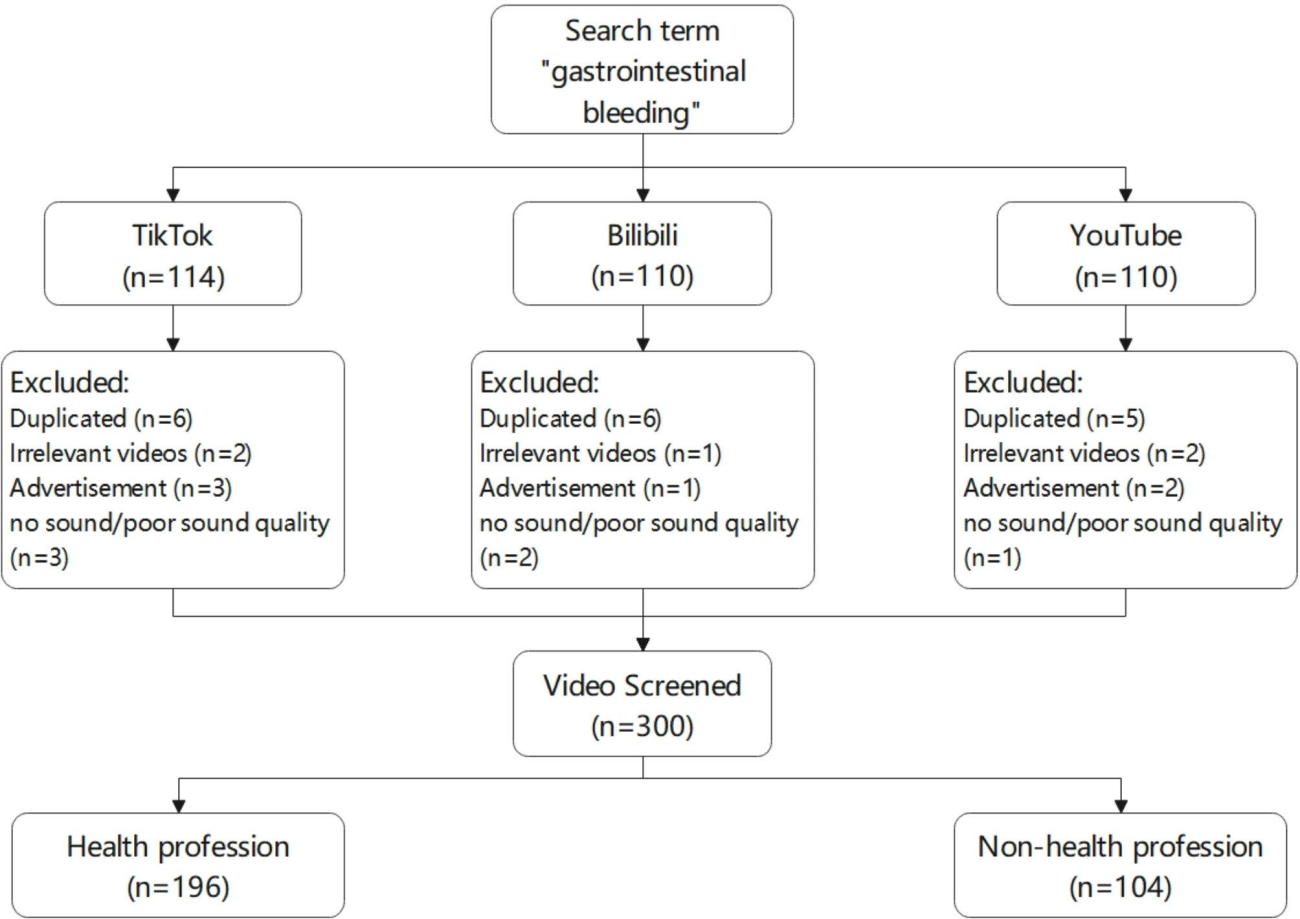
Flowchart of patients included in this study.

All collected videos were sourced from three video platforms: Bilibili, TikTok, and YouTube. Using “gastrointestinal bleeding” as the keyword, we searched and selected the top 100 videos based on comprehensive rankings on TikTok, Bilibili, and YouTube. This specific term was chosen due to its high relevance and common usage in medical contexts, ensuring that the search results would be focused and comprehensive. The term “gastrointestinal bleeding” is widely recognized and used by both health professionals and the general public, making it an appropriate and effective search term for this study. Before conducting the searches, we logged out of all accounts and cleared search histories to avoid biases from personalized recommendations. The video retrieval was conducted on June 12, 2024, and included videos uploaded before June 5, 2024, as recent videos may have unstable view and like counts that do not accurately reflect viewer engagement. Figure 1 illustrates the detailed process of video selection. This flowchart provides a visual representation of the steps taken, starting from the initial search to the final selection of videos for analysis. Each video’s content and detailed information were independently evaluated and recorded by two investigators, Jingsong Wang and Bingxi Liu, both of whom hold master’s degrees and are licensed physicians. If discrepancies arose between the investigators, they resolved them through discussion and consultation with the corresponding author. The exclusion criteria were as follows: (1) videos unrelated to the topic; (2) duplicate videos; (3) videos used for advertising or commercial purposes; (4) videos with incomplete data; (5) videos with no sound/poor sound quality.

### Video characteristics

We systematically extracted and recorded various characteristics of the videos, including views, likes, comments, favorites, shares, duration, upload date, source of upload, video content, GQS, JAMA, and Modified DISCERN scores. The duration of each video was measured using the platform’s built-in video player, which displays the total length of the video in seconds. Based on the uploader’s account name, verification status, and the information presented in the videos, they were categorized into two main groups: health profession and non-health profession. The non-health profession group included news agencies, profit organizations, non-health nonprofit organizations, science communicators, and general users. The health profession group included health professionals (such as doctors and nurses) and health institutions (such as public hospitals).

In this study, we used three commonly applied standardized scales to evaluate the video content: GQS, JAMA, and Modified DISCERN. These scales were employed to assess and analyze the quality and effectiveness of the videos. The GQS is a widely used tool for evaluating the quality of health information in videos, assessing dimensions such as quality, fluency, comprehensiveness, and usefulness to patients (Supplementary Table 1). It uses a 5-point scale ranging from 1 (poor quality) to 5 (excellent fluency and quality)^25–28^. The JAMA score evaluates the reliability of the videos based on four key criteria: (1) authorship disclosure; (2) listing of copyright information and references, along with content sources; (3) provision of the initial date and subsequent updates; and (4) disclosure of conflicts of interest, funding, sponsorship, advertising support, and video ownership (Supplementary Table 2). The total score is 4, with each criterion scoring 1 point^29–31^. The Modified DISCERN score is a widely validated and applied tool that evaluates videos based on five questions: (1) Is the video clear, concise, and understandable? (2) Are the information sources reliable? (3) Is the information provided balanced and unbiased? (4) Does it provide additional sources of information for patients? (5) Does it appropriately address areas of uncertainty or controversy? Each affirmative answer scores 1 point, while each negative answer scores 0 points^32–34^ (Supplementary Table 3). Additionally, we assessed the comprehensiveness of video content across six major aspects: definition, symptoms, risk factors, tests, treatment, and outcomes. Based on the extent to which the video content met these six criteria, videos were classified into five categories: no content (0 points), little content (0.5 points), some content (1 point), most content (1.5 points), and extensive content (2 points).

### Statistical analysis

In this study, data analysis was conducted using SPSS version 27.0 (IBM, Chicago, IL, USA) and R statistical software version 4.3.1. For quantitative data that followed a normal distribution, results were expressed as mean ± standard deviation (x ± SD) and analyzed using t-tests. For non-normally distributed data, results were expressed as median and interquartile range (IQR) and analyzed using the Mann-Whitney U test. Categorical variables were presented as frequencies and percentages, and differences were analyzed using the chi-square test or Fisher’s exact test. A two-tailed p-value of less than 0.05 was considered statistically significant.

## Results

### Video characteristics

**Fig. 2:**
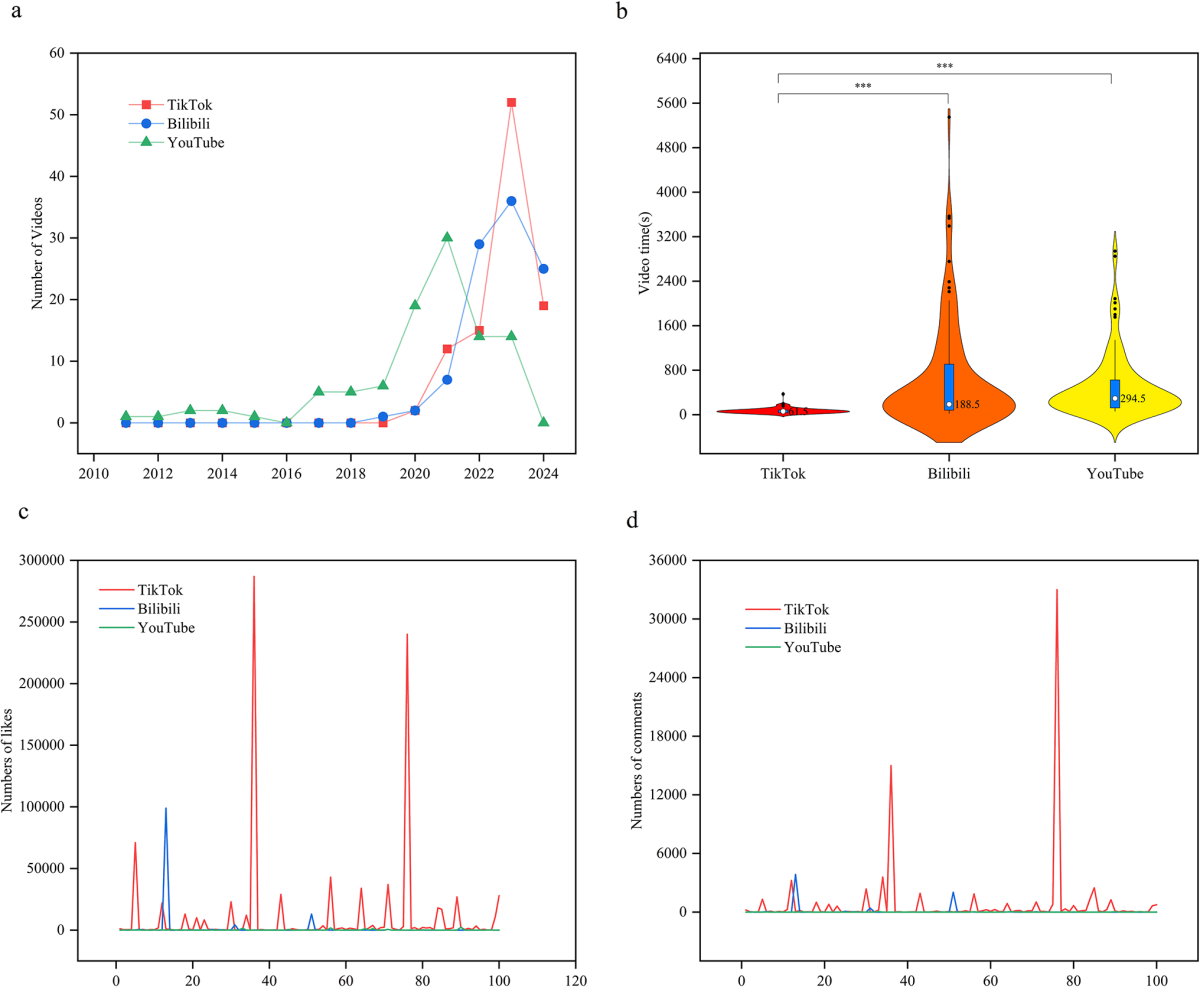
General information on gastrointestinal bleeding-related videos from three video platforms. (**a**) Gastrointestinal bleeding-related videos published on various video platforms between 2010 and 2024. (**b**) Viewing times of gastrointestinal bleeding-related videos on three video-sharing platforms. (**c**) Number of likes on three video-sharing platforms. (**d**) Number of comments on three video-sharing platforms.

A total of 300 videos related to gastrointestinal (GI) bleeding were selected from Bilibili, TikTok, and YouTube for further analysis. These videos were uploaded between August 9, 2011, and June 5, 2024. The majority of videos on Bilibili, TikTok, and YouTube were uploaded between 2020 and 2024 (Fig. 2a). YouTube had the longest span of video uploads, showing a statistically significant difference compared to Bilibili and TikTok (*p* < 0.01) (Table 2).

**Table 1:** Comparison of source and scores of videos about Gastrointestinal bleeding on three different platforms.

Parameters	Total	TikTok	Bilibili	YouTube	*P*
Video source					<0.01
For-profit organizations	6(2.0%)	1(1%)	3(3%)	2(2%)	
Non-health nonprofit organizations	17(5.7%)	2(2%)	2(2%)	13(13%)	
News agencies	32(10.7%)	2(2%)	5(5%)	25(25%)	
General users	22(7.3%)	1(1%)	16(16%)	5(5%)	
Science communications	27(9.0%)	3(3%)	21(21%)	3(3%)	
Health professionals	141(47.0%)	89(89%)	40(40%)	12(12%)	
Health institutions	55(18.3%)	2(2%)	13(13%)	40(40%)	
GQS					<0.01
1 score	19(6.3%)	13(13%)	2(2%)	4(4%)	
2 score	91(30.3%)	45(45%)	29(29%)	17(17%)	
3 score	118(39.3%)	28(28%)	45(45%)	45(45%)	
4 score	57(19.0%)	12(12%)	16(16%)	29(29%)	
5 score	15(5.0%)	2(2%)	8(8%)	5(5%)	
JAMA					<0.01
1 score	38(12.7%)	10(10%)	21(21%)	7(7%)	
2 score	130(43.3%)	52(52%)	43(43%)	35(35%)	
3 score	118(39.3%)	34(34%)	30(30%)	54(54%)	
4 score	14(4.7%)	4(4%)	6(6%)	4(4%)	
Modified DISCERN					<0.01
1 score	22(7.3%)	10(10%)	10(10%)	2(2%)	
2 score	94(31.3%)	37(37%)	30(30%)	27(27%)	
3 score	150(50.0%)	50(50%)	39(39%)	61(61%)	
4 score	29(9.7%)	3(3%)	17(17%)	9(9%)	
5 score	5(1.7%)	0(0%)	4(4%)	1(1%)	

**Table 2:** Basic index of videos about Gastrointestinal bleeding on three different platforms.

	TikTok	Bilibili	YouTube	*P*
Views	/	561.5(213.5-2668.5)	1321.5(236.5-7077.5)	0.627
Number of likes	612.5(249.0-2099.0)	7.0(2.0–45.0)	8.5(1.0-61.5)	<0.01
Number of comments	62.0(22.5–227.0)	0(0–3)	0(0-1.5)	<0.01
Number of collections	129.0(36.0-468.5)	6.0(1.0–30.0)	/	0.447
Number of favorites	198.5(49.5-695.5)	4.0(0.5–12.0)	/	0.181
Duration (s)	61.5(37.5–85.5)	188.5(82.5-908.5)	294.5(122.0-625.5)	<0.01
Time since upload (d)	298.0(185.0-562.0)	395.5(166.5-689.5)	1126.5(840.0-1509.0)	<0.01

**Fig. 3:**
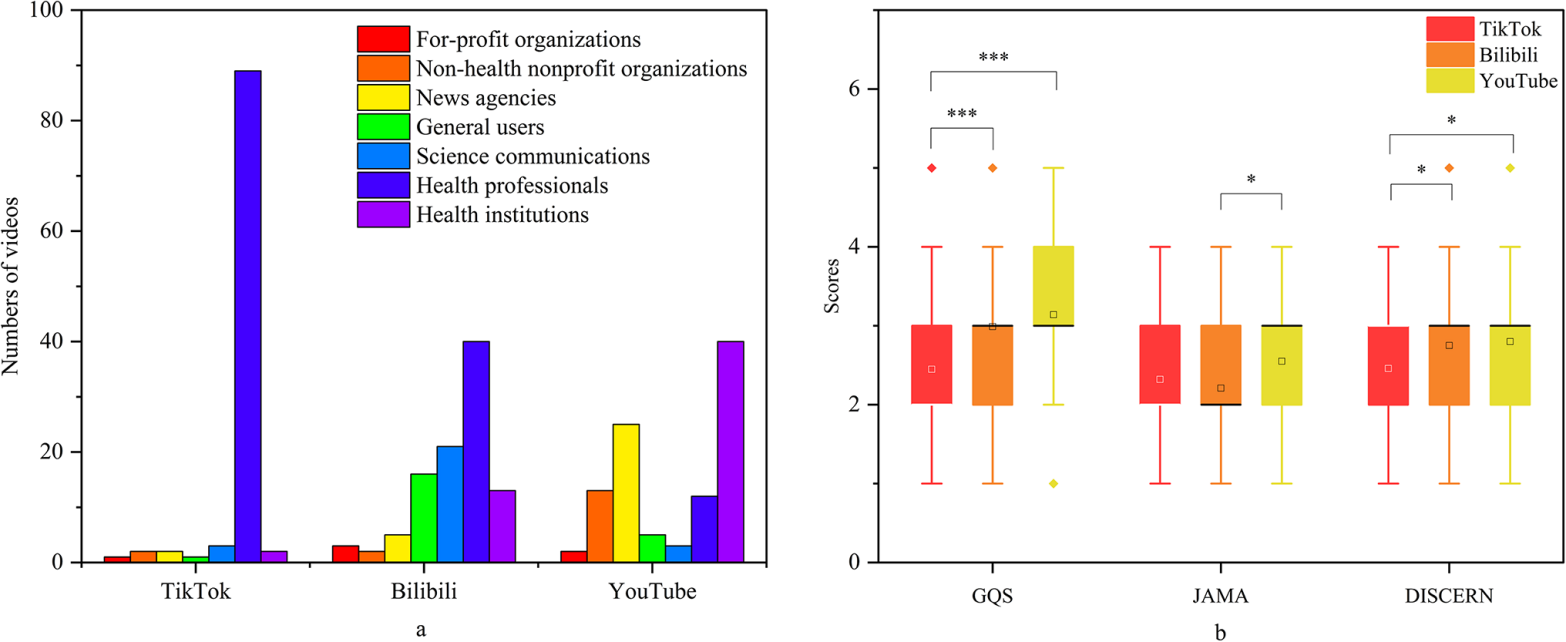
(**a**) Video sources for the three platforms. (**b**) Comparison of GQS, JAMA, and Modified DISCERN scores on three social platforms.

Of the 300 videos, 196 (65.3%) were uploaded by sources within the health profession group, while the remaining 104 (34.7%) were from non-health profession sources (Table 3). Among the health profession group, videos were primarily uploaded by health professionals (141 videos, 47%) and health institutions (55 videos, 18.3%). In the non-health profession group, news agencies were the largest contributors with 32 videos (10.7%), followed by science communicators (27 videos, 9.0%), general users (22 videos, 7.3%), non-health nonprofit organizations (17 videos, 5.7%), and profit organizations (6 videos, 2.0%) (Table 1; Fig. 3a).

**Table 3:** Comparison of video features on different video source.

	Health profession	Non-health profession	*P*
Likes	66.5(3.5–512.0)	26.5(4.0-113.0)	<0.05
Comments	4.5(0-54.5)	1.0(0-7.5)	<0.05
Duration (s)	92.0(59.0-183.5)	280.0(125.0-703.0)	<0.01
GQS	3.0(2.0–4.0)	3.0(2.0–4.0)	<0.01
JAMA	2.0(2.0–3.0)	2.0(1.5-3.0)	<0.01
DISCERN	3.0(2.0–3.0)	2.0(2.0–3.0)	<0.01

On Bilibili and TikTok, most videos were uploaded by health professionals, totaling 40 and 89 videos, respectively. YouTube was dominated by videos from health institutions, with 40 videos. The duration of the 100 GI bleeding videos on TikTok ranged from 7 to 372 s, with a median duration of 188.5 s. In contrast, the duration of the 100 videos on Bilibili ranged from 22 to 5350 s, also with a median of 188.5 s. On YouTube, the median duration of 100 videos was 61.5 s (IQR 37.5–85.5) (Table 2). There was a statistically significant difference in video durations between TikTok and Bilibili (IQR 82.5-908.5) and YouTube (IQR 122.0-625.5) (*p* < 0.001) (Fig. 2b).

During the study period, TikTok videos received significantly more likes and comments compared to Bilibili and YouTube, with medians of 62.0 (IQR 22.5–227.0) and 129.0 (IQR 36.0-468.5), respectively, and the differences were statistically significant (*p* < 0.05) (Table 2; Fig. 2). There was no significant difference in video views between Bilibili and YouTube (*p* > 0.05), with medians of 561.5 (IQR 213.5-2668.5) and 1321.5 (IQR 236.5-7077.5), respectively. Additionally, there was no statistical difference in the number of favorites and shares between TikTok and Bilibili.

Further comparison of video sources revealed that videos uploaded by health professionals had higher median likes (66.5, IQR 3.5–512.0) and comments (4.5, IQR 0-54.5) compared to those from non-health professionals (likes: 26.5, IQR 4.0-113.0; comments: 1.0, IQR 0-7.5), with statistically significant differences (*p* < 0.05) (Table 3). However, videos from the health profession group were shorter in duration (median: 92.0, IQR 59.0-183.5) compared to those from non-health profession sources (median: 280.0, IQR 125.0-703.0), with a significant difference (*p* < 0.01).

#### Video content analysis

**Fig. 4:**
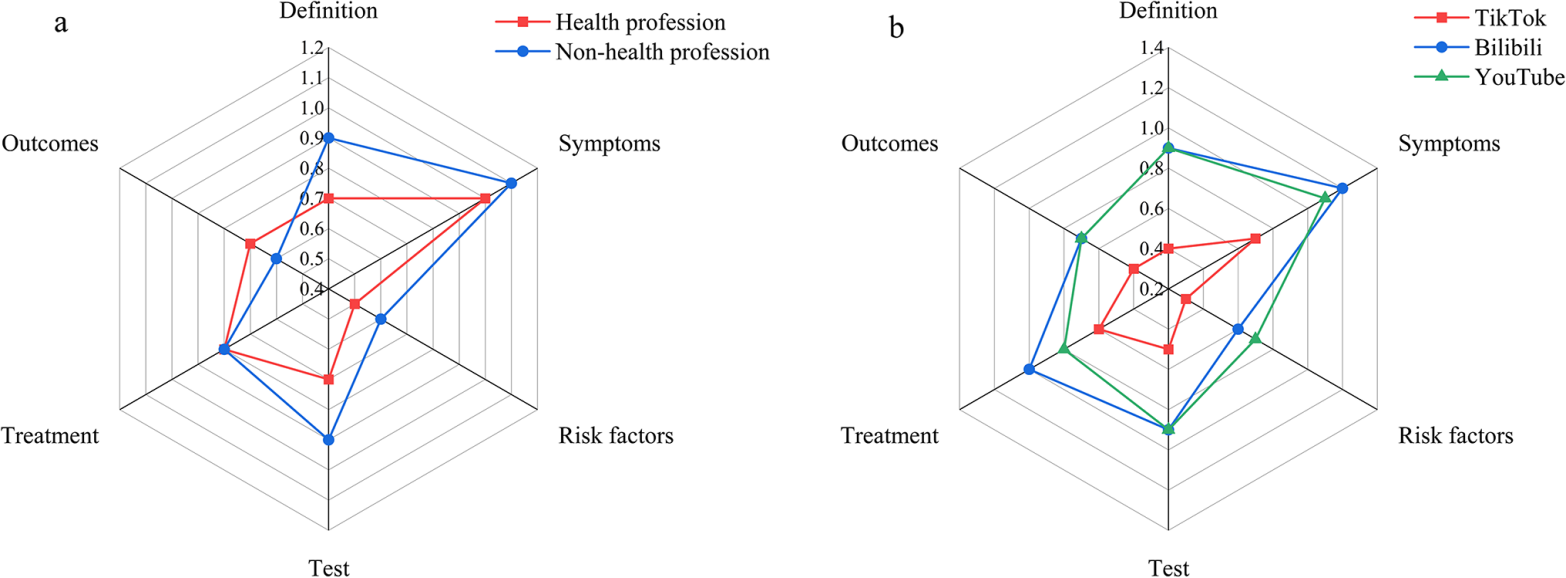
(**a**) Comparison of content comprehensiveness between health professionals and non-health professionals. (**b**) Comparison of content comprehensiveness on different platforms.

To evaluate the completeness and comprehensiveness of different videos, this study assessed six aspects: definition, symptoms, risk factors, test, treatment, and outcomes. Overall, the results showed that few videos provided comprehensive information on gastrointestinal (GI) bleeding, with most videos focusing more on describing the symptoms (Table 4). Specifically, 12.3% of the videos included extensive content related to symptoms, followed by 9.3% covering diagnosis, 9% covering outcomes, 7.7% covering definitions, and 4.3% covering risk factors. Content related to treatment and risk factors was less frequently mentioned, comprising 4.3% and 3.8% of the videos, respectively (Fig. 4).

**Table 4:** Completeness of video content.

Video content	Definition	Symptoms	Risk factors	Test	Treatment	Outcome
No content (0 points)	82(27.3%)	27(9%)	129(43%)	86(28.7%)	69(23%)	114(38%)
Little content (0.5 points)	60(20%)	64(21.3%)	61(20.3%)	63(21%)	67(22.3%)	57(19%)
Some content (1 point)	105(35%)	131(43.7%)	81(27%)	90(30%)	89(29.7%)	85(28.3%)
Most content (1.5 points)	30(10)	41(13.7%)	16(5.3%)	33(11%)	48(16%)	27(9%)
Extensive content (2 points)	23(7.7%)	37(12.3%)	13(4.3%)	28(9.3%)	27(9%)	17(5.7%)

**Fig. 5:**
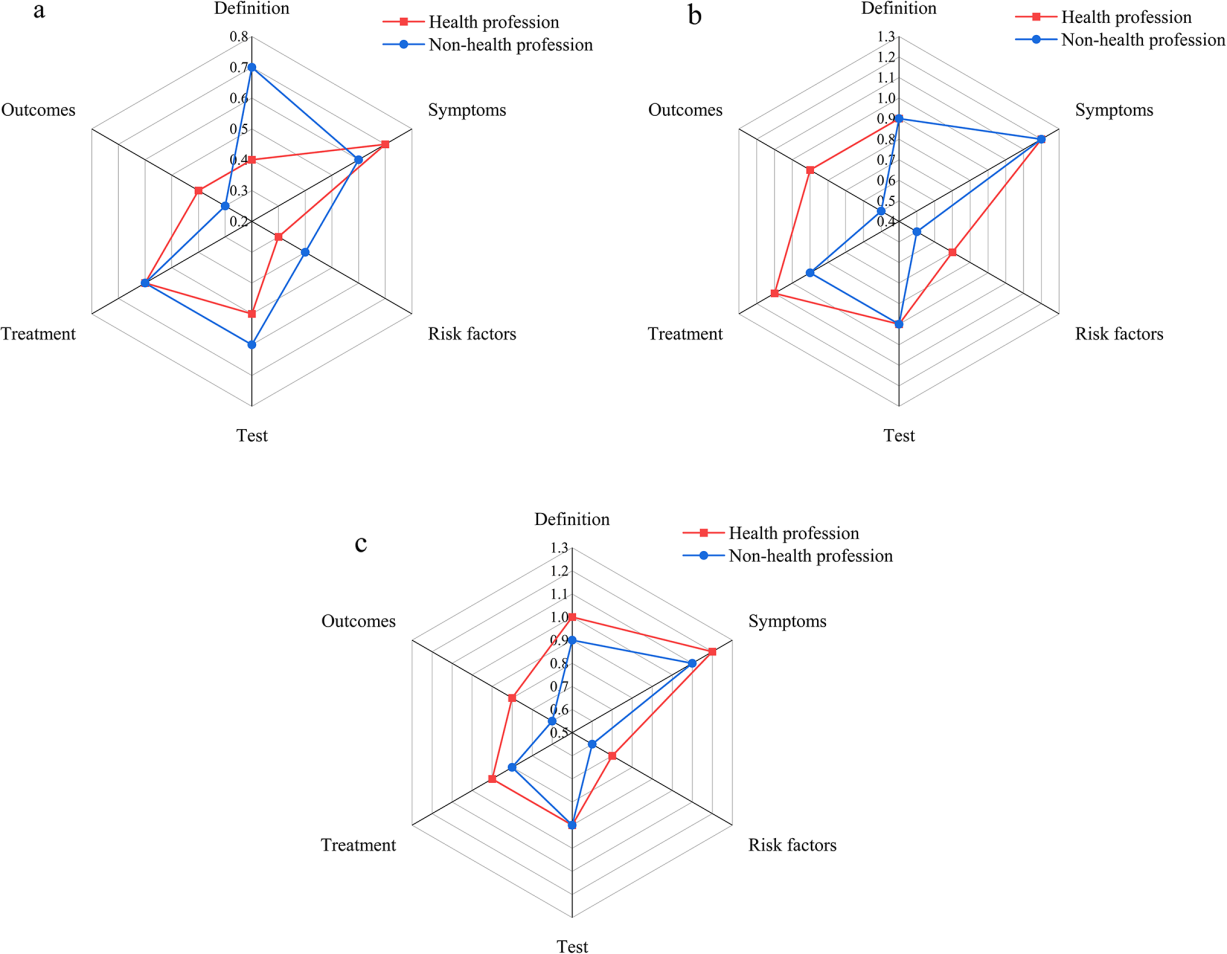
Comparison of content comprehensiveness in the health professional and non-health professional domains across different video platforms. (**a**) TikTok; (**b**) Bilibili; (**c**) YouTube.

When analyzed based on the source of the videos, those uploaded by health professionals generally contained more information on definitions, symptoms, risk factors, tests, and treatment compared to videos from non-health professionals. Analyzing the videos from the three different platforms, TikTok videos were found to cover significantly less information on GI bleeding definitions, symptoms, risk factors, tests, treatment, and outcomes compared to those from Bilibili and YouTube, which did not show a significant difference between each other. In a subgroup analysis of the platforms, videos from the health profession group on Bilibili and YouTube included more comprehensive content across all aspects than those from the non-health profession group (Fig. 5). However, on TikTok, there was no significant difference in the content comprehensiveness between videos from health professionals and non-health professionals.

### Video quality and reliability evaluation

The videos collected from the three different platforms were scored based on their respective evaluation criteria. The overall scores for the videos were: GQS score of 3 (IQR: 2–3), JAMA score of 2 (IQR: 2–3), and DISCERN score of 3 (IQR: 2–3) (Fig. 3). Statistical analysis showed that, compared to TikTok, videos on Bilibili and YouTube generally received higher GQS and Modified DISCERN scores, with these differences being statistically significant. Additionally, there were significant differences in JAMA scores between videos on Bilibili and YouTube (Fig. 3).

## Discussion

Gastrointestinal (GI) bleeding is a common and serious clinical issue in gastroenterology, particularly prevalent among the elderly population. It can be caused by various conditions, including peptic ulcers, esophageal varices, and tumors. Despite significant advancements in the diagnosis and treatment of GI bleeding, its management and prognosis remain challenging^1,5,7^. A patient’s level of awareness and understanding of GI bleeding is a crucial factor affecting the incidence, treatment outcomes, and prognosis of the condition. Enhancing public knowledge about GI bleeding, including its potential causes, symptoms, diagnosis, and treatment, can help patients recognize bleeding symptoms at an early stage, seek medical attention promptly, adhere better to treatment plans, and reduce recurrence rates.

High-quality medical education can effectively convey essential information about GI bleeding, enabling patients to understand the risk factors, preventive measures, and necessary lifestyle management skills, thereby significantly improving treatment outcomes. However, due to limited medical resources, many GI bleeding patients are unable to receive detailed disease education or care plans from clinical professionals^4,35^. Consequently, obtaining disease-related information via the internet has become a vital aspect of current health education.

The rapid development of social media and video-sharing platforms provides an accessible and convenient means for disseminating medical information. These platforms, such as Bilibili, TikTok, and YouTube, offer a wide range of content that can reach a broad audience^36,37^. However, the quality of information varies significantly, posing a risk of misinformation. This study highlights the importance of evaluating and improving the quality of GI bleeding-related videos on these platforms to ensure that accurate and comprehensive medical knowledge is available to the public.

Short video platforms have become popular internet tools whose significance in modern society cannot be overlooked. These platforms enable users to quickly access the latest and comprehensive information. Research has shown that health-related short videos on TikTok have garnered over 1.7 million likes and 176,000 comments, indicating that short videos have gradually become a primary means for patients to obtain health-related information^38,39^. However, the quality of these videos varies widely, and some may even disseminate incorrect health information, posing potential risks to public health and leading to the deterioration of patients’ conditions. Currently, there are no studies that specifically evaluate the quality of GI bleeding videos on platforms such as TikTok, Bilibili, and YouTube. Therefore, it is crucial to review video content and filter out high-quality information to ensure its effectiveness in patient health education and disease self-management.

This study conducted a statistical analysis of GI bleeding-related videos on TikTok, Bilibili, and YouTube, exploring differences in metrics such as likes, comments, duration, and video quality. Additionally, the study compared videos from health professional and non-health professional sources. The statistical results indicated that GI bleeding-related videos on TikTok received significantly more likes and comments compared to those on Bilibili and YouTube (*p* < 0.05). This may be due to TikTok’s broader user base and higher interactivity, with its recommendation algorithm favoring highly interactive videos. However, the average duration of TikTok videos was significantly shorter than those on Bilibili and YouTube (*p* < 0.05), consistent with TikTok’s focus on short videos. While short videos can quickly capture viewers’ attention, their depth and information content are often insufficient, limiting the conveyance of complex and detailed information.

Regarding video quality, TikTok videos scored significantly lower on the GQS and JAMA scales compared to those on Bilibili and YouTube (*p* < 0.05). Figure 3a shows that the comprehensiveness of TikTok video content is significantly lower than that of YouTube and Bilibili videos. This indicates that although TikTok videos have higher interactivity, their scientific accuracy, reliability, and comprehensiveness are clearly lacking. In contrast, videos on Bilibili and YouTube are longer, more detailed, and comprehensive, resulting in better quality scores.

Further analysis revealed that videos uploaded by health professionals received significantly higher scores in likes, comments, GQS, JAMA, and DISCERN compared to those from non-health professional sources. This suggests that videos produced by health professionals have advantages in content quality, information reliability, and viewer recognition. These videos typically contain more professional medical knowledge, detailed explanations, and scientific evidence, making them more popular and trusted by viewers. However, despite the higher quality of videos from health professionals, their content comprehensiveness did not significantly differ from that of videos from non-health professionals. This phenomenon is particularly evident on TikTok. Although the number of videos from health professionals on TikTok is higher, the comprehensiveness and depth of some of these videos are not as good as those from non-professional sources. This may be due to the overall lower quality of TikTok videos, the short video format limiting the conveyance of complex information, and health professional uploaders favoring short videos to attract attention, resulting in no significant content difference between professional and non-professional videos.

Overall, while videos published by health profession tend to be more numerous and of higher quality compared to those from non-health sources, the general quality of videos related to gastrointestinal (GI) bleeding is not satisfactory. The disparity between high-quality and low-quality information is due to both objective and subjective factors within healthcare, making it difficult for the public to discern accurate information in the complex social media environment. Several reasons may explain this phenomenon: First, the format of short video platforms limits the depth and comprehensiveness of content. Platforms like TikTok, which focus on short video formats, make it challenging for creators to explain complex medical knowledge and case analyses in detail within a limited timeframe. Despite health profession’s extensive expertise, it is difficult to fully showcase this knowledge in short videos, leading to insufficient content depth and comprehensiveness.Second, platform recommendation algorithms prioritize video interactivity (such as likes and comments) over scientific accuracy and quality. This algorithmic bias can result in high-quality, highly professional videos being overshadowed by low-quality but highly interactive videos in the recommendation feed. Consequently, higher-quality GI bleeding videos may struggle to gain adequate exposure and attention in the competitive content environment, affecting their dissemination effectiveness. Third, viewer preferences and demands also influence video quality and content. Many viewers on video platforms seek quick, concise information rather than in-depth medical knowledge. This demand drives creators to produce shorter, more easily understandable videos, often at the expense of content professionalism and comprehensiveness. This trend is particularly evident in medical topics like GI bleeding, which require detailed explanations and professional knowledge.

Therefore, improving the information quality of short videos is crucial for maintaining patient health and reducing disease incidence. To enhance the overall quality of GI bleeding-related videos, we recommend the following: first, platforms should optimize their recommendation algorithms to increase the weight of high-quality, professionally strong videos, ensuring that these videos gain more exposure and recommendations, while reducing the recommendation frequency of low-quality, highly interactive but scientifically inaccurate videos. Second, platforms should strengthen the review process for medical and health-related video content to ensure the scientific accuracy, reliability, and authority of the content, thereby reducing the risk of patients being misled. Finally, more experienced healthcare professionals should be encouraged to publish medical videos related to diseases to improve the content and quality of these videos.

## Limitations

This study has several limitations. First, it focuses on Chinese video content and does not include videos in other languages, which may limit the applicability of the results to non-Chinese video content. Second, the evaluation of video quality in this study is confined to three major video platforms; the quality of videos on other platforms requires further investigation. Lastly, due to platform restrictions and data limitations, metrics such as view counts, favorites, and shares for some platform videos were difficult to obtain in this study. Future studies could consider expanding the scope to include more languages and a broader range of platforms to provide a more comprehensive understanding of the quality and content of GI bleeding-related videos.

## Conclusion

In summary, this study provides reliable and valuable information for the public to understand the current state of gastrointestinal (GI) bleeding-related videos on social media platforms. The results indicate that the content and quality of short videos related to GI bleeding are not satisfactory, with many low-quality videos still prevalent on these platforms. The differences in content comprehensiveness and depth between videos from health professionals and those from non-health professionals are not significant, highlighting the need for further improvement in video quality. There is a need for more high-quality short videos related to GI bleeding, along with greater participation from health professionals. Additionally, platforms should strengthen the management and supervision of related videos to accurately disseminate relevant knowledge to patients. Moreover, platforms should optimize their recommendation algorithms to increase the weight of high-quality, professionally strong videos, ensuring that these videos gain more exposure and recommendations. This will help in disseminating accurate and high-quality information about GI bleeding to the public.

## Electronic supplementary material

Below is the link to the electronic supplementary material.

Supplementary Material 1

Supplementary Material 2

Supplementary Material 3

## Data Availability

The data sets generated during and/or analysed during this study are available from the corresponding author on reasonable request.
