# Supplementary material for: Assessing the content and quality of GI bleeding information on Bilibili, TikTok, and YouTube: a cross-sectional study
Source: Sci Rep. 2025 Apr 28;15:14856. doi: 10.1038/s41598-025-98364-7 (PMC12038001; doi:10.1038/s41598-025-98364-7)
Supplement: Supplementary file 1 — Supplementary Material 1 [file 41598_2025_98364_MOESM1_ESM.docx]

Supplementary table 1. Global Quality Score (GQS) benchmark criteria.

| Score | Global Score Description |
| --- | --- |
| 1 score | poor quality, poor traffic, most information missing, not of any use to the patient |
| 2 score | Generally poor quality with poor flow, some information listed but many important topics lacked, of very limited use to patients |
| 3 score | medium quality with suboptimal flow, some of the main information was fully discussed but other information discussed insufficiently, somewhat helpful to patients. |
| 4 score | Good quality and generally goo flow, most of the relevant information is listed, but some topics not covered, useful to patients. |
| 5 score | Excellent quality and excellent flow, very useful for patients |
