# Supplementary material for: Assessing the content and quality of GI bleeding information on Bilibili, TikTok, and YouTube: a cross-sectional study
Source: Sci Rep. 2025 Apr 28;15:14856. doi: 10.1038/s41598-025-98364-7 (PMC12038001; doi:10.1038/s41598-025-98364-7)
Supplement: Supplementary file 2 — Supplementary Material 2 [file 41598_2025_98364_MOESM2_ESM.docx]

Supplementary table 2. The Journal of the American Medical Association (JAMA) benchmark criteria.

| Score* | Score component | |
| --- | --- | --- |
| 1 score | Authorship | Provided authorship information |
| 1 score | Attribution | Listed copyright information and references/sources |
| 1 score | Currency | Included the initial date and subsequent updates |
| 1 score | Disclosure | Disclosed any potential conflicts of interest, funding, sponsorship, advertising support or video ownership |

*The criteria of each aspect were scored separately, and 1 point was accumulated when the criteria were reached. A total reliability score ranging from 0 to 5 was obtained.
