# Supplementary material for: Assessing the content and quality of GI bleeding information on Bilibili, TikTok, and YouTube: a cross-sectional study
Source: Sci Rep. 2025 Apr 28;15:14856. doi: 10.1038/s41598-025-98364-7 (PMC12038001; doi:10.1038/s41598-025-98364-7)
Supplement: Supplementary file 3 — Supplementary Material 3 [file 41598_2025_98364_MOESM3_ESM.docx]

Supplementary table 3. Modified DISCERN benchmark criteria.

| Score* | Reliability Score |
| --- | --- |
| 1 score | The video was clear, concise and understandable |
| 1 score | The information sources were reliable |
| 1 score | The information presented was balanced and unbiased |
| 1 score | Additional sources of information were provided for patient reference |
| 1 score | Areas of uncertainty or controversy were appropriately addressed. |

*The criteria of each aspect were scored separately, and 1 point was accumulated when the criteria were reached. A total reliability score ranging from 0 to 5 was obtained.
